# Undifferentiated high-grade pleomorphic sarcoma of the colon: a rare case report and literature review

**DOI:** 10.1186/s12876-022-02189-x

**Published:** 2022-03-10

**Authors:** Xu Han, Linxian Zhao, Yu Mu, Guoliang Liu, Guohong Zhao, Hongyu He, Shu Wang, Jiannan Li

**Affiliations:** 1 Department of General Surgery, The Second Hospital of Jilin University, Changchun, 130041 Jilin China; 2 Operating Theater and Department of Anesthesiology, The Second Hospital of Jilin University, Changchun, 130041 Jilin China; 3 Department of Radiotherapy, The Second Hospital of Jilin University, Changchun, 130041 Jilin China

**Keywords:** Undifferentiated pleomorphic sarcoma, Malignant fibrous histiocytoma, Case report, Colon

## Abstract

**Background:**

Undifferentiated pleomorphic sarcoma (UPS), also known as malignant fibrous histiocytoma (MFH), hardly originates from the colorectum.

**Case presentation:**

We reported a 65-year-old female presented with UPS in the descending colon. Computed tomography (CT) revealed an irregularly thickened descending colon. On colonoscopy examination, an ulcerative tumour was identified. The patient received radical resection of the left colon and partial enterectomy. The resected tumor was ulcerative, 10 cm × 8 cm × 5 cm in size, and infiltrated the serosa layer. Postsurgical pathology showed that the tumor was high-graded UPS in the colon with large amounts of necrotic tissues.

**Conclusions:**

UPS in the large intestine is a rare malignant tumor with a poor prognosis and unknown pathogenesis. The main treatment for UPS is early complete resection. Postsurgery adjuvant radiotherapy or chemotherapy can be attempted.

## Background

Sarcomas are heterogeneous malignant tumors originating from the mesenchymal tissues, only accounting for 1% of malignancies in adults [1]. Undifferentiated pleomorphic sarcoma (UPS), also known as malignant fibrous histiocytoma (MFH), accounts for 28% of all soft-tissue sarcomas and usually occurs in the extremities and retroperitoneum [2, 3]. Currently, the pathogenesis of UPS is not completely understood. However, it has been proposed that some predisposing factors are involved in the occurrence of UPS, including genetic abnormalities, chemoradiotherapy stimulation, chronic irritation, and lymphedema [4]. In addition, UPS is more common in male patients aged between 60 and 80 [5]. UPS is more aggressive with strong regional invasiveness and distant metastasis. It has been reported that the prognosis of UPS is poor due to late diagnosis and a lack of effective treatments [6]. Especially, the prognosis of intra-abdominal UPS is poorer than those in the extremities [6]. However, UPS in the large intestine is extremely rare. In this study, we report a rare case of high-grade UPS in the colon.

## Case presentation

This study was approved by the Institutional Review Board and the Ethics Committee of the Second Hospital of Jilin University, Changchun, China. Informed consent was obtained for the publication of this case. The relevant medical details are represented in Table 1.

**Table 1:** Relevant medical details

Medical history	1. Hypertension for about 10 years, with the highest blood pressure of 180/100 mmHg
	2. Cerebral infarction for 10 years with no obvious sequelae
	3. Coronary heart disease for about 1 year
Major complaints	1. Fever and fatigue for 1 week with the highest body temperature of 39.4 °C
	2. Bloody stool, abdominal distention, and decreased exhaustion and defecation 1 month earlier
	3. The abdominal distention increased gradually during recent 1 month
Physical examination	1. Pale eyelids, thickened breath sounds of both lungs, and a drum sound in the abdomen through abdominal perfusion
	2. A 5 cm × 5 cm sized mass could be touched in the left abdomen with mild tenderness
Biochemical examination	White blood cells (29.3 × 10^9^/L, normal: 3.5–9.5 × 10^9^/L); carbohydrate antigen 125 (CA125, 49.3 U/mL, normal: 0–35 U/mL); hemoglobin (75 g/L, normal: 115–150 g/L)
Computer tomography (CT)	Bilateral pleural effusion, pericardial effusion, pelvic fluid, and an irregularly thickened wall of the descending colon
Colonoscopy	An ulcerative tumor in the descending colon, which invaded the wall of the descending colon circularly

A 65-year-old female came to the Respiratory Department of the Second Hospital of Jilin University due to fever and fatigue for 1 week with the highest body temperature of 39.4 °C. The patient denied any symptoms of cough, expectoration, chest tightness, or shortness of breath. The body temperature of the patient still ranged from 38.0 to 39.0 °C after taking oral anti-inflammatory drugs. The relevant medical details of the patient are summarized in Table 1, including medical history, major complaints, physical examination, biochemical results, computer tomography (CT) examination, and colonoscopy.

**Fig. 1:**
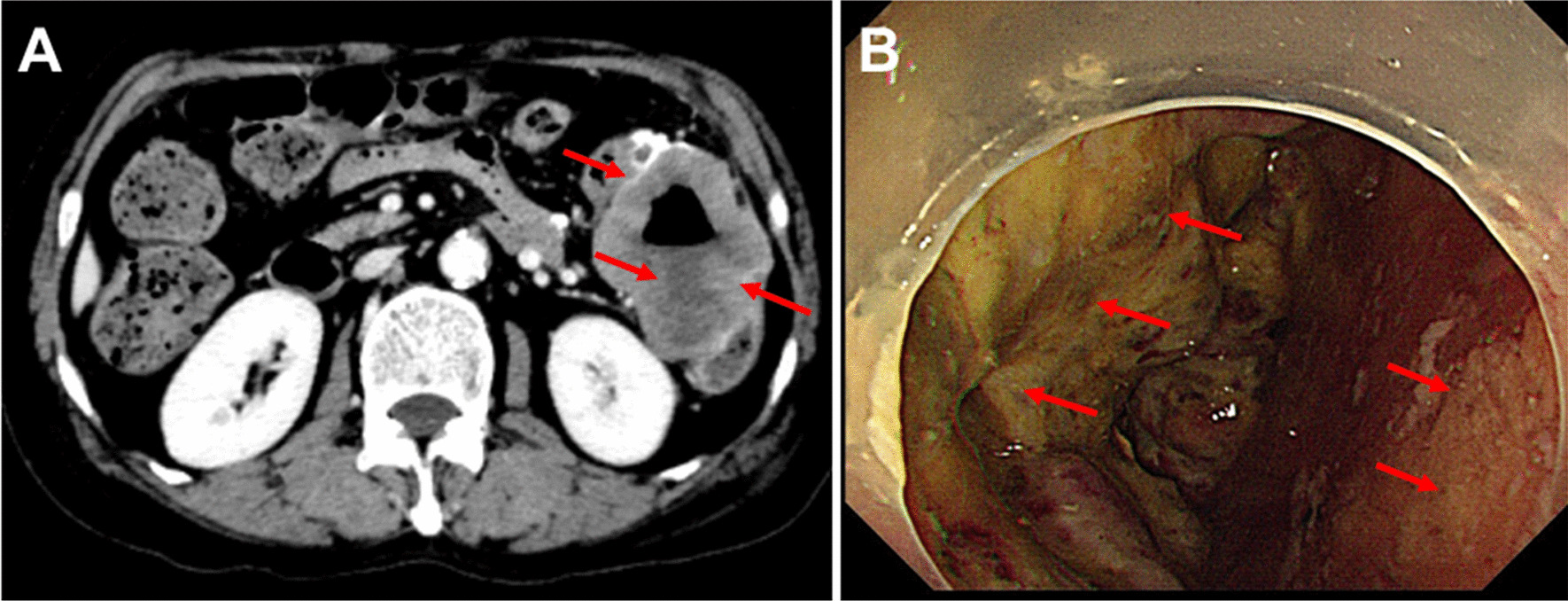
Abdominal CT and electric colonoscopy examinations. **A** Abdominal CT examination indicates the tumor (red arrows) in the descending colon. **B** Colonoscopy shows the ulcerative tumor (red arrows), which has invaded the descending colon wall

CT imaging revealed an irregularly thickened descending colon (Fig. 1A). The patient received anti-infective, antipyretic, and other symptomatic supportive treatments. During colonoscopy examination, an ulcerative tumor was found in the descending colon (Fig. 1B), which obstructed further colonoscopy examination. No pathological biopsy was performed during colonoscopy due to the Aspirin medication history of the patient. Antiplatelet therapy was usually reckoned as a contraindication for biopsy in our local guidelines because endoscopists believe that antiplatelet therapy can increase bleeding risk during this procedure [7].

**Fig. 2:**
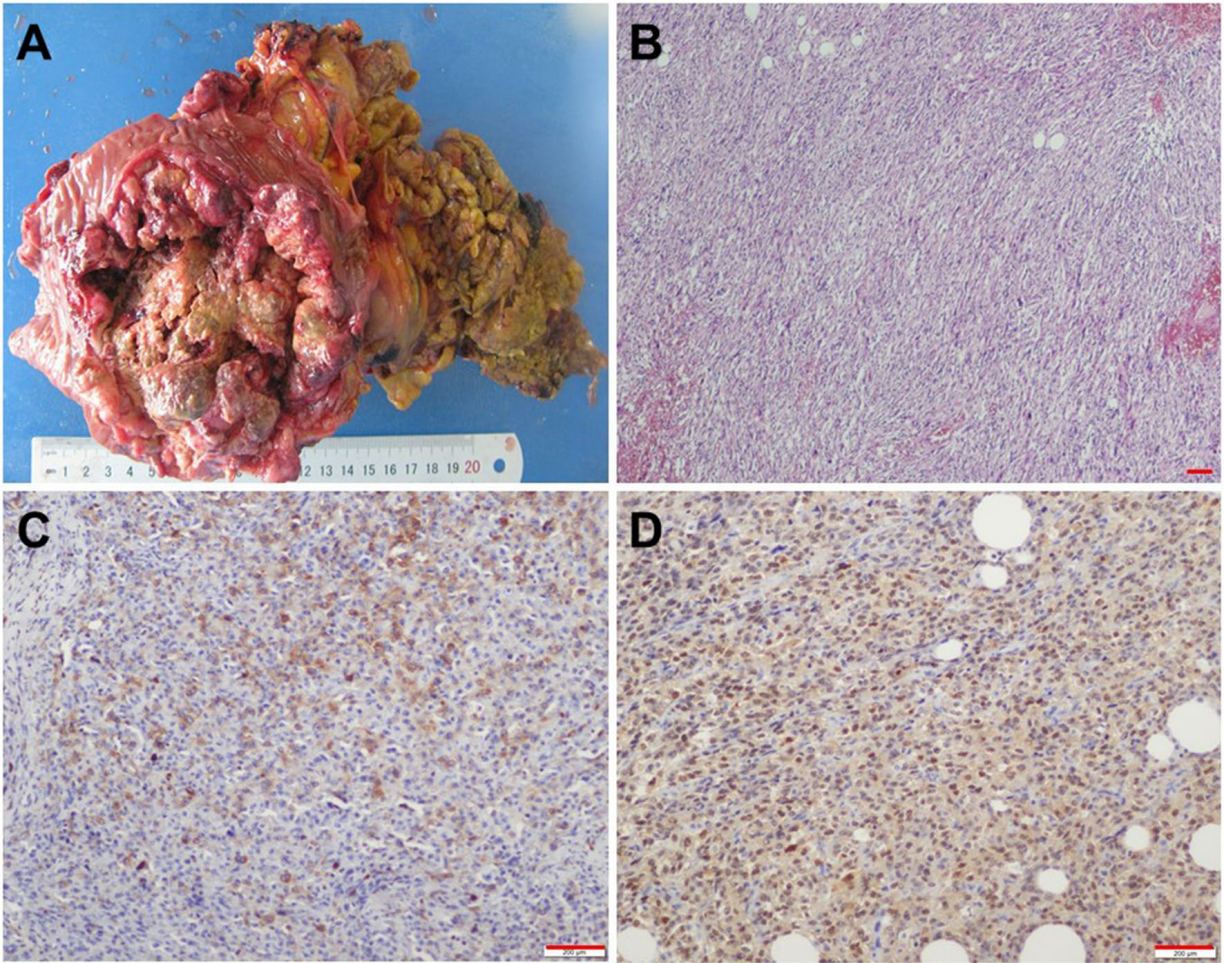
Pathological examination and immunohistochemical staining of the tumor. **A** The resected tumor is ulcerative, 10 cm × 8 cm × 5 cm in size, and has infiltrated into the serosa layer. **B** Histopathology shows that the tumor is high-graded UPS in the colon with fibrous hyperplasia, necrotic tissues, and inflammatory cells. **C** Immunohistochemical examination shows that tumor cells are positive for α1-antichymotrypsin. **D** Immunohistochemical examination shows that tumor cells exhibit marked positivity for vimentin. Scale bars = 200 µm

The symptoms in the lung of the patient eased gradually after symptomatic treatment and the body temperature was normalized. The patient was transferred to the Department of General Surgery for laparotomy. An ulcerative tumor in the splenic curvature of the colon was found during the surgery. The tumor invaded the small intestine, which was 10 cm away from the ligament of Treitz. The patient received radical resection of the left colon, and the transverse colon and sigmoid colon were anastomosed end to end. Furthermore, the small intestine and its mesangium invaded by the tumor were dissected, and the small intestines on both sides were anastomosed end-to-end. The resected tumor was ulcerative, 10 cm × 8 cm × 5 cm in size, and infiltrated in the serosa layer (Fig. 2A). Microscope CX31 (Olympus, Japan) and the Microscopic Image Analysis Software 11.0 were used for histopathological analysis. The measured resolution for all the microscopic images was 0.5 nm. Postsurgical pathology showed that the tumor was high-graded UPS in the colon with large necrotic tissues (Fig. 2B). The serosa layer of the colon was filled with fibrous hyperplasia, vessel hyperplasia, inflammatory cell infiltration, and necrotic tissues. The mucous layer of the colon was filled with inflammatory cells. Further immunohistochemical analysis showed that the tumor cells were positively stained for α1-antichymotrypsin (Fig. 2C) and vimentin (Fig. 2D), which was consistent with the characteristics of UPS. The patient recovered well and was discharged from our department on the 10th-day after surgery and received no further treatment. One year follow-up was performed, and CT and colonoscopy did not reveal any signs of local recurrence or distant metastasis.

## Discussion and conclusions

First found and named by Ozzello et al*.* in 1963 [8], MFH includes four histological subtypes: myxoid, inflammatory, storiform-pleomorphic, and giant-cell [9]. In 2002, the World Health Organization redefined the MFH classification. The subtypes of storiform-pleomorphic MFH, giant-cell MFH, and inflammatory MFH were classified into fibrous histiocytic tumors and replaced by UPS and UPS with giant cells. Another subtype of myxoid MFH was defined as the myofibroblastic category and renamed to myxofibrosarcoma [10].

In order to further explore UPS, we searched the literature on PubMed, updated to May 01, 2021, using keywords including “Undifferentiated pleomorphic sarcoma,” “malignant fibrous histiocytoma,” “colon,” “rectum,” and their variants. Studies were included per the following inclusion criteria: (1) clinical features of the patient available; (2) primary UPS of the colon or rectum and confirmed by histology; (3) case reports. Studies with duplicate data or data not relevant to UPS were excluded. After the screening, only 20 cases were included (Table 2) [11–30]. The characteristics of UPS were analyzed in this study.

**Table 2:** The UPS of colorectum in the literature

Author	Age (year)	Sex	Tumor location	Longitude diameter (cm)	Symptoms	Surgery	Adjuvant therapy	Follow-up
Sewell et al. [11]	74	M	Transverse colon	8.5	Anorexia	Yes	No	12 months
					Diarrhea			No recurrence or metastasis
Levinson and Tsang [12]	17	M	Transverse and sigmoid colon	10, 8	Abdominal pain	Yes	NA	NA
Rubbini et al. [13]	60	M	Sigmoid colon	7	Bloody stool	Yes	Chemotherapy	53 months
								Dead, liver metastasis
Baratz et al. [14]	73	M	Transverse colon	15	Anorexia	Yes	No	6 months
					Anemia			No recurrence or metastasis
Waxman et al. [15]	52	F	Sigmoid colon	7.5	Abdominal pain	Yes	No	9 months
								Dead, local recurrence
Satake and Matsuyama [16]	62	M	Ascending and transverse colon	17, 19	Abdominal mass	No	NA	NA
Katz et al. [17]	62	F	Cecum	2	Abdominal pain	Yes	No	3 months
								No recurrence or metastasis
Murata et al. [18]	50	M	Ascending colon	9.5	Abdominal distention	Yes	Chemotherapy	10 months
								No recurrence or metastasis
Huang and Wei [19]	12	M	Ascending colon	3.5	Abdominal pain	Yes	No	16 months
								No recurrence or metastasis
Makino et al. [20]	72	M	Transverse colon	7	Abdominal pain	Yes	No	4 months
								Dead, local recurrence
Hiraoka et al. [21]	64	M	Cecum	5	Abdominal distention	Yes	No	4 months
								Dead, lymph nodes metastasis
Udaka et al. [22]	47	M	Ascending colon	7	Abdominal mass	Yes	No	13 months
								No recurrence or metastasis
Gupta and Malani [23]	46	F	Cecum and ascending colon	17	Abdominal distention	Yes	No	36 months
					Abdominal mass			No recurrence or metastasis
Okubo et al. [24]	66	M	Ascending colon	14.5	Abdominal pain	Yes	No	33 months
								No recurrence or metastasis
Kawashima et al. [25]	50	F	Descending colon	10	Abdominal pain	Yes	No	7 years
								No recurrence or metastasis
Ji et al. [26]	68	F	Ascending colon	8	Fever	Yes	Radiotherapy	5 years
								Dead, local recurrence
Bosmans et al. [27]	73	M	Sigmoid colon	3.5	Anemia	Yes	No	22 months
								No recurrence or metastasis
Wang et al. [28]	55	M	Sigmoid colon	6.0	Abdominal pain	Yes	No	5 months
								Dead, local recurrence
Fu et al. [29]	70	M	Cecum	12	Abdominal pain	Yes	No	1 month
								Dead, lung metastasis
Singh et al. [30]	55	M	Rectum	2.5	Perineal pain	Yes	Chemotherapy	46 months
							Radiotherapy	No recurrence or metastasis

M, male; F, female; NA, not applicable

According to the studies in Table 2 and the case in this study, UPS in the colorectum mainly occurs in male patients with a male to female ratio of 2.5:1. The patients' age ranges from 12 to 74 years, with an average of 56.81 ± 16.65. Although UPS could occur in any part of the colorectum, only one case was reported to be originated from the rectum. Most cases were diagnosed as a large tumor with a 2–19 cm diameter, with a median of 7.75 cm. The main symptoms of UPS originating from the large intestine include abdominal pain, abdominal distention, anorexia, diarrhea, anemia, fever, and perineal pain.

UPSs in the colon are mesenchymal tumors. Therefore, most of UPSs originate from deep fascia or muscularis and grow out of the intestinal lumen [27]. Usually, clinical manifestations of UPSs in the colon are not completely specific. In this case, the main clinical presentations are similar to colorectal cancer, include bloody stools, fatigue, and reduced bowel movement frequency, which may be caused by tumor hemorrhage and inevitable tumor outgrow. In addition, due to the intact of the colonic mucosa, nothing abnormal can be observed in colonoscopy even UPSs in the colon have already occurred. However, if the tumor is sufficiently large and has infiltrated the mucosa layer, a colonoscopy examination is useful. For example, in this case, colonoscopy indicated an ulcerative tumor that prevented further colonoscopy examination. To date, the diagnosis of UPS in the colon remains highly challenging due to the lack of effective early cancer screening strategies [26]. Histopathology is still the gold standard for UPS diagnosis. Microscopically, the histological characteristics of UPS include the complexity of cell components, pleomorphism of tumor cells, and the diversity of tissue structure [27]. Tumor tissues often include fibroblasts, histone cells, giant cells, xanthoma cells, and inflammatory cells [27]. Although immunohistochemical stains could be useful for UPS diagnosis, no reproducible immunophenotype or protein expression can be used in more specific subclassification [31]. More specifically, some special staining can be used to exclude other tumors. For example, the pleomorphic liposarcoma is positive for SMA, S-100 protein, keratins, and desmin, while the pleomorphic leiomyosarcoma and pleomorphic rhabdomyosarcoma are only positive for desmin [31]. However, UPSs are frequently positive for vimentin, actin, CD68, α1-antitrypsin, and α1-antichymotrypsin [31].

Radical surgery is the primary treatment for UPS in the colorectum. However, UPS in the colorectum often has exogenous growth that infiltrates the surrounding tissues. Therefore, extensive or radical excision will not prevent possible local recurrence or distant metastasis. The effects of postsurgical chemotherapy or radiotherapy are still unclear. Among the 20 cases in earlier studies and the 1 case in this study, 20 patients received radical surgery, 2 patients received postsurgical chemotherapy, 1 patient received postsurgical radiotherapy, and 1 patient received postsurgical chemotherapy and radiotherapy. Of the 19 patients followed up after surgery, 7 had local recurrences or distant metastasis. The follow-up time was generally short, and the follow-up data of some patients were lost. The 6 months, 1 year, 2 years, and 5 years survival rates of patients with UPS in the large intestine were 77.78% (14/18), 75.00% (12/16), 63.64% (7/11), and 12.50% (1/8), respectively.

UPS in the colorectum is a rare malignant tumor with a poor prognosis and unknown pathogenesis. Nearly half of the patients with UPS died of postoperative recurrences or metastasis. The primary treatment for UPS is early complete resection of the tumor. Adjuvant radiotherapy and/or chemotherapy can be attempted after the surgery with individual efficacy.

## Data Availability

All data generated or analyzed are included in this published article.

## Abbreviations

UPS: Undifferentiated pleomorphic sarcoma
MFH: Malignant fibrous histiocytoma
CT: Computed tomography
CEA: Carcinoembryonic antigen
